# Autonomic Responses to Emotional Stimuli in Children Affected by Facial Palsy: The Case of Moebius Syndrome

**DOI:** 10.1155/2019/7253768

**Published:** 2019-04-08

**Authors:** Ylenia Nicolini, Barbara Manini, Elisa De Stefani, Gino Coudé, Daniela Cardone, Anna Barbot, Chiara Bertolini, Cecilia Zannoni, Mauro Belluardo, Andrea Zangrandi, Bernardo Bianchi, Arcangelo Merla, Pier Francesco Ferrari

**Affiliations:** ^1^Unit of Neuroscience, Department of Medicine and Surgery, University of Parma, Parma, Italy; ^2^Deafness and Neural Plasticity Lab, School of Psychology-University of East Anglia, Norwich, UK; ^3^Institut des Sciences Cognitives Marc Jeannerod UMR 5229, CNRS, and Université Claude Bernarde Lyon, Bron Cedex, France; ^4^Infrared Imaging Lab ITAB-Institute of Advanced Biomedical Technologies and Department of Neuroscience, Imaging and Clinical Sciences, University “G. D'Annunzio” Chieti-Pescara, Italy; ^5^Unit of Audiology and Pediatric Otorhinolaryngology, University Hospital of Parma, Parma, Italy; ^6^Clinical Neuropsychology, Cognitive Disorders and Dyslexia Unit, Department of Neurology, Arcispedale Santa Maria Nuova-IRCCS, Reggio Emilia, Italy; ^7^NeXT: Neurophysiology and Neuroengineering of Human-Technology Interaction Research Unit, Campus Bio-Medico University, Rome, Italy; ^8^Maxillo-Facial Surgery Division, Head and Neck Department, University Hospital of Parma, Parma, Italy

## Abstract

According to embodied simulation theories, others' emotions are recognized by the unconscious mimicking of observed facial expressions, which requires the implicit activation of the motor programs that produce a specific expression. Motor responses performed during the expression of a given emotion are hypothesized to be directly linked to autonomic responses associated with that emotional behavior. We tested this hypothesis in 9 children (*M*_age_ = 5.66) affected by Moebius syndrome (MBS) and 15 control children (*M*_age_ = 6.6). MBS is a neurological congenital disorder characterized by underdevelopment of the VI and VII cranial nerves, which results in paralysis of the face. Moebius patients' inability to produce facial expressions impairs their capacity to communicate emotions through the face. We therefore assessed Moebius children's autonomic response to emotional stimuli (video cartoons) by means of functional infrared thermal (fIRT) imaging. Patients showed weaker temperature changes compared to controls, suggesting impaired autonomic activity. They also showed difficulties in recognizing facial emotions from static illustrations. These findings reveal that the impairment of facial movement attenuates the intensity of emotional experience, probably through the diminished activation of autonomic responses associated with emotional stimuli. The current study is the first to investigate emotional responses in MBS children, providing important insights into the role of facial expressions in emotional processing during early development.

## 1. Introduction

Moebius syndrome (MBS) is a rare congenital syndrome affecting approximately 1 in 50000 to 1 in 500000 live births [1], with no gender predominance [2]. The disorder presents with varying phenotypes and severity and is characterized by unilateral or bilateral facial paralysis, as well as impaired bilateral movement of the eyes. This is due to maldevelopment of the VI and VII cranial nerve nuclei early in prenatal life [3–7]. The VI and VII cranial nerves control, respectively, the abduction of the eyes and the muscles used to generate facial expressions, lip speech, and eye closure. V, IX, X, and XII cranial nerves can also be affected [8–10]. Other congenital abnormalities are sometimes associated with the syndrome, including sensorineural hearing loss, craniofacial malformations, limb anomalies, Poland syndrome (underdevelopment of the pectoralis muscle and hand malformation), hypoglossia, and poor coordination [10, 11]. Most patients are of normal intelligence, while approximately 9-15% present mild mental retardation, and another 0-5% are diagnosed with autistic-like behaviors [12–14].

One of the most prominent features of MBS patients is their inability to smile or produce any facial movement, which limits their capacity to communicate emotions through the face [15–19].

Evidence has shown that the motor component of emotional facial expressions is associated with an involuntary autonomic nervous system (ANS) response [20]. It has been proposed that the coding of emotional stimuli in macaque monkeys is mediated by the activity of brain networks including both cortical motor and specific limbic regions [21]. Human neuroimaging studies have demonstrated that, in addition to motor regions, the observation and direct experience of an emotion activate specific brain areas (i.e., the anterior insula, the anterior cingulate cortex (ACC), and the amygdala) [22], which are important not only in the control of the motor components of emotions but also in orchestrating the complex visceromotor responses associated with an emotional state (increase/decrease in heart rate (HR), changes in blood pressure, pupil dilation, piloerection, metabolic changes, etc.) [21, 23–25]. Emotional processing therefore relies on a complex network of brain regions in which some structures, such as the insular cortex, the amygdala, and the ACC, could coordinate the autonomic responses typical of the limbic system with the motor modifications associated with the expression of an emotion [26, 27]. This tight connection between motor and autonomic responses is therefore of utmost importance when investigating disturbances involving the motor commands controlling emotional expressions.

Several studies posit that the same motor regions involved in the generation of a particular facial expression of emotion are also implicated in recognizing that emotion in others [28–30]. The neuronal basis of this process is underpinned by a mirror mechanism, implemented by a parietal-premotor cortical network known as the “mirror neuron system” (MNS) [31, 32]. The MNS in humans has been proposed to support not only the understanding of others' action intentions [33–35] but also the recognition of others' emotions through activation in the observer of a neural motor representation similar to that expressed by the observed individual [25–27, 34–40]. Emotion recognition therefore occurs via unconscious mimicking of the observed expression, which requires the implicit activation of those motor programs responsible for the production of a particular facial expression and associated physiological responses (also named *reverse simulation model*) [41–44]. According to embodied simulation theories [41, 45–49], the perception of an emotional facial expression is accompanied by the simulation of that specific emotional state in the motor, somatosensory, affective, and reward systems of the perceiver [44, 50, 51].

In light of these premises, facial motor impairment in MBS patients could impact several processes related to emotions. A few studies have shown that adult Moebius patients can recognize others' emotions to some degree, but the results are mixed. This is likely due to certain methodological limitations including patient sample size, lack of clinical evaluation, nonobjective assessment (i.e., self-evaluation), and variations in the measures and tasks used [52–55]. In addition, previous studies have centered on adults, who may have developed alternative strategies throughout their lifespan in order to cognitively recognize facial expressions. These supportive strategies, whereby specific facial cues of emotion expression (e.g., the mouth corners turned up or down) are extracted [56, 57], could have positively affected their ability to discern different emotions later in life. Finally, the above-mentioned studies focused on Moebius patients' emotion recognition abilities without investigating the autonomic component of emotional processing.

Bearing in mind that the motor and autonomic components associated with an emotional expression interact with each other, one could say that the congenital absence of facial muscle activity and relative proprioceptive feedback could result in a dysfunctional autonomic response to emotional stimuli and difficulties in recognizing others' emotions [52, 55]. MBS patients therefore represent an interesting population to investigate this.

The measurement of the autonomic component of emotional processing during childhood would enable the constraints linked to cognitive processing of emotional information to be bypassed. In this sense, the lack of facial expressivity in Moebius children makes them an ideal subgroup to study emotional processing during the early phases of development, when complex cognitive strategies have yet to emerge.

We hypothesized that the lack of facial motor activity in MBS children during the decoding of emotions could induce an altered autonomic response while watching emotional videos, as well as difficulties in deciphering emotional facial stimuli. To this end, we monitored participants' autonomic response during observation of emotional stimuli using functional infrared thermal (fIRT) imaging, a dynamic and noninvasive method of measuring skin temperature distribution [58]. Facial skin thermal patterns depend on subcutaneous vessels transporting blood heat. These vessels regulate blood flow via local vascular resistance (vasodilation and vasoconstriction) and arterial pressure [59]. Therefore, by recording the dynamics of facial cutaneous temperature, it is possible to assess ANS activity and infer the subject's emotional state [60–64].

fIRT has been shown to be effective in detecting several affective states, including extreme stress [63], startle [65], fear [66], arousal [67], and happiness [68]. For example, fear experienced during a threatening and distressing situation [62, 69–71], as well as the experience of stress [71, 72] or guilt [61], is related to a decrease in nasal tip temperature due to subcutaneous adrenergic vasoconstriction [73]. On the contrary, social interaction [62, 74] and sexual arousal [75] produce an increase in nasal tip temperature, caused by the vasodilation effect of the parasympathetic nervous system on the autonomic state of the individual. Crucially, due to its low invasiveness and versatility, fIRT results are particularly suitable for use with younger individuals, as well as clinical populations [61, 62, 76, 77].

In the present study, we expected to observe a weaker thermal modulation in MBS participants compared to control subjects, and we hypothesized that the motor impairment of MBS patients would result in an impaired autonomic response during emotion observation.

## 2. Materials and Methods

### 2.1. Participants

We recruited 9 children (5 males) with MBS aged 4 to 8 years (mean age 5.66, SD = 1.78). Moebius participants exhibited unilateral or bilateral facial paralysis, as well as related neurological symptoms (see Table 1); all were referred to as cognitively able in the study, and all were attending mainstream schools at a level appropriate to their age. Moebius children were recruited through the clinical center at the University of Parma, which specializes in the diagnosis of MBS and therapeutic intervention. Only patients without cognitive disability or diagnosis of autism were included in the experiments. We also recruited 15 healthy children (control group) (9 males) in the same age range (mean age 6.6, SD = 1.79). All participants were informed that they would be videotaped by means of a thermal camera and a webcam. All parents gave their informed written consent after full explanation of the procedure, which is in accordance with the 1964 Declaration of Helsinki. The study was approved by the Ethics Committee of the University of Parma.

### 2.2. Materials

Thermal IR imaging was performed by means of a digital thermal camera FLIR T450sc (IR resolution: 320 × 240 pixels; spectral range: 7.5–13.0 *μ*m; thermal sensitivity/NETD: <30 mK at 30°C). The frame rate was set to 5 Hz (5 frames/sec). A remote-controlled webcam (Logitech webcam C170) was used to film the participants' behavior, so as to record their level of attention while watching video stimuli.

### 2.3. Procedure and Stimuli

**Figure 1:**
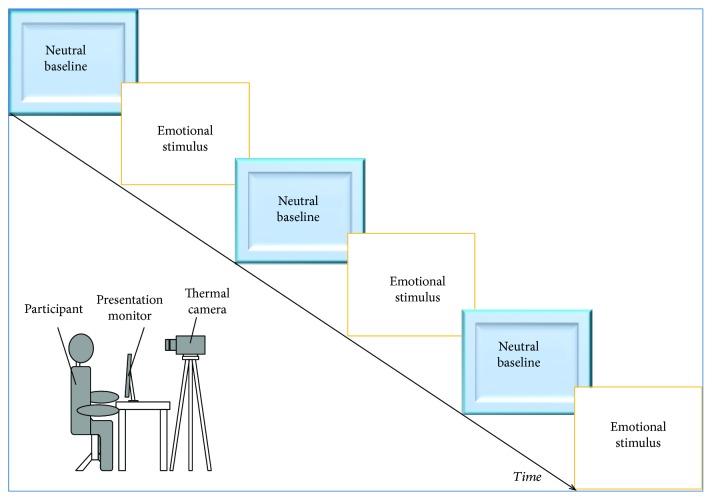
Experimental paradigm. Schematic overview of the experimental paradigm.

Prior to testing, each participant was left to acclimatize for 10 minutes to the experimental room and to allow their skin temperature to stabilize. The recording room was set at a standardized temperature (23°C) and humidity (50–60%) and was not subject to direct sunlight, ventilation, or airflow. During an initial neutral interaction, the experimenter asked the child to answer questions related to personal data (e.g., name and age). The child was then invited to watch a series of video stimuli displayed on a computer monitor (32.5 × 22.7 cm) placed 60 cm far from the chair where the child was sitting. According to other thermal imaging studies using video stimuli [60, 78, 79], our sequences included 6 different video clips (neutral baseline-happiness-neutral baseline-sadness-neutral baseline-fear), with each emotional video preceded by a neutral video. Stimuli were comprised of short clips taken from the Internet in which the main character of the scene was in a happy, sad, or scary situation. The emotional video clips varied in their duration (mean = 81.38 sec; SD = 43.49), while neutral video clips (ones with no emotional content) lasted about 30 sec (mean = 28.83 sec; SD = 3.69) (Figure 1). Chosen stimuli represented the kind of videos that children of this age are familiar with.

Video clips were validated before the experiment in order to ensure that they were easily comprehensible and represented the specific emotion deemed appropriate for the age range of interest here. To do this, we presented neutral (baseline), happy, sad, and scary videos to a separate group of 16 children (8 males) with a mean age of 7.5 years; participants were asked to categorize the video clip as evoking feelings of “happiness,” “sadness,” “fear,” or “neutral baseline.” The average percentage of correct recognition was 95.83%. Based on our validation study, we randomly presented two video sequences from a list of six. The choice to present two sequences only was based on expected fatigue, habituation, and difficulty in sustaining children's attention for long periods of time.

During the experimental session, thermal and video cameras were placed above the monitor, one meter away from the participant. Cameras were automatically calibrated and manually fixed to capture a frontal view of the child's face. Facial thermal images and videotapes were recorded during each video presentation.

**Figure 2:**
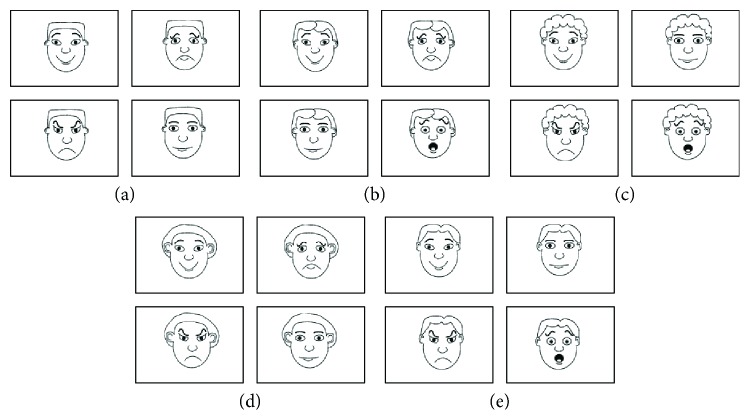
Example of cartoon pictures presented during TEC-1 (component I, emotion recognition).

At the end of each short video clip, participants were asked a series of questions concerning (1) the emotional state of the main characters depicted in the video cartoon and (2) the child's own emotional involvement while watching each video clip. Unfortunately, in most cases, the children did not reply to the questions and therefore it was not possible to apply statistical analyses. To overcome the difficulty of this assessment, we also administered the Italian standardized version (see [80]) of the *Test of Emotion Comprehension* (TEC-1) [81], so as to obtain an index of the individual's capacity to discriminate different emotions. MBS children were administrated component I of the TEC-1, which assesses emotion recognition by means of facial expression discrimination (Figure 2). Four simple drawings were presented on an A4 sheet of paper, which included four out of five possible emotions depicted by cartoon facial expressions. The children were asked to indicate which of the facial expressions was happy, sad, angry, scared, or “just alright” (i.e., neutral component). Figure 2 illustrates the items used to assess children's emotion recognition. Five successive items were used to test children's recognition of emotions. Depending on the participant's own gender, a corresponding version of the drawings (i.e., female or male) was presented. Component I of the TEC-1 was also used to test the emotion recognition ability in 15 healthy subjects in the same age range as Moebius participants. The full experimental session, including both emotional sequences and TEC-1, lasted a maximum of 45 minutes.

### 2.4. Data Analysis

**Figure 3:**
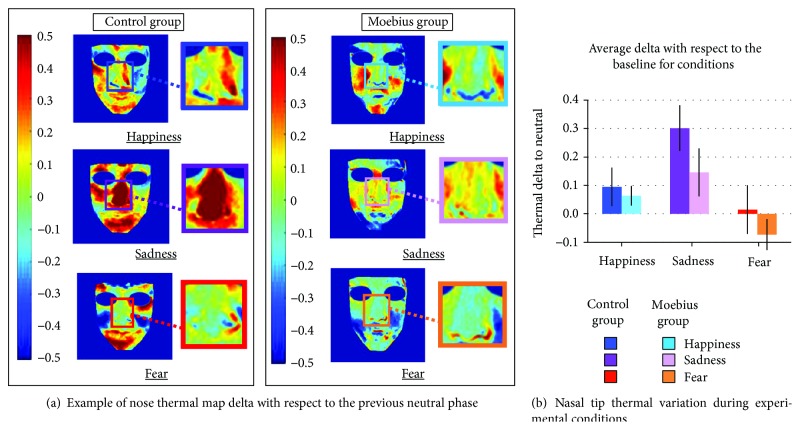
(a) Thermal modulation in an example control participant and Moebius patient during the “happiness,” “sadness,” and “fear” conditions. In the figure, the inlays present the entire nasal area, but elliptic nasal tip ROIs were used for analyses (*A* = 297 pixels; MajorAxisLength = 20.35 pixels; MinorAxisLength = 18.64 pixels) [62, 65]. The control participant shows stronger thermal variation during the sadness condition than the Moebius patient. (b) Mean temperature values during each of the experimental conditions, baseline-corrected with respect to the neutral condition. Both control and Moebius participants show a significant nasal tip temperature increase during the “sadness” condition (^∗^*p* ≤ 0.001). Means and standard errors (SE) are reported for each condition in both control and Moebius groups.

A quantitative analysis was carried out to measure temperature variations of participants' nasal tips. Elliptic regions of interest (ROIs) with identical shape and dimensions (*A* = 297 pixels; MajorAxisLength = 20.35 pixels; MinorAxisLength = 18.64 pixels) were utilized. We focused on this ROI for two main reasons. First, given the relatively low incidence of MBS, the specific age sample of interest, and the pioneering nature of the current study, we decided to include patients with unilateral or bilateral facial paralysis. The nasal tip is a nonlateralized ROI, so its temperature should not be modulated by the lateralization of nerve impairment. Second, the nasal tip has been shown to be particularly sensitive to emotional state transitions [62, 65, 70, 82]. This area of the face is indeed highly innervated by adrenergic fibers, resulting in a privileged window on a participant's autonomic state. More specifically, sympathetic nervous responses to emotional and distressing stimulation produce a decrease in nasal tip temperature whereas parasympathetic responses result in a temperature increase in this ROI [62, 65, 68, 71, 77, 82, 83]. Thermal signals were extracted through the use of the software Morphing GUI, developed with customized MATLAB algorithms (The Mathworks Inc., Natick, MA). This analysis procedure is more extensively described in [84]. Due to the high computational load associated with the morphing procedure, we decided to downsample the collected dataset. Given the slow nature of thermal responses, such a processing choice did not affect the precision of temperature change detection [62, 84]. For each video stimulus presented to the child, three thermal images were extracted (one frame at the beginning, one in the middle, and one at the end of each video) and morphed. These particular frames were selected in order to minimize the effect of the respiratory cycle on the thermal imprinting of the subject [71]. The three frames selected within each condition (emotional or neutral) were averaged. In order to interpret any affective response, the selection of an appropriate baseline represents the starting point for defining the directionality of the physiological change during emotional arousal [76]. For this reason, we selected video clips with no emotional content (see Procedure and Stimuli) to eliminate the interindividual variability in the subjects' temperature and to minimize the effect of participants' circadian variations on our data. We followed a typical procedure for thermal data analysis [62]: subtraction of the mean thermal value of each neutral condition from the mean thermal value of its following experimental condition (happiness, sadness, and fear). In this way, we obtained a dataset of thermal variation for each emotional condition relative to the neutral condition. The thermal variations for the two trials belonging to the same condition were then averaged to obtain a mean value for each emotion (happiness, sadness, and fear) (see Figure 3(a)). This was used as the variable of interest in our statistical analyses, including the comparison of MBS participants and the control group.

During TEC-1 administration, participants' answers were noted on the answer sheet by the experimenter and subsequently coded (1 point for each correct answer and 0 for each wrong answer).

### 2.5. Statistical Data Analysis

A repeated measures ANOVA (6 × 2) was performed on the neutral baseline temperature values in order to confirm that baseline temperature did not significantly differ between the Moebius and control groups (*p* > 0.05). A repeated measures ANOVA (3 × 2) was performed on the mean nasal tip values (emotion compared to neutral baseline) for all participants [76]. The emotion condition (happiness, sadness, and fear) was set as a within-subjects factor, while the group (Moebius and controls) was set as a between-subjects factor [85, 86]. Bonferroni post hoc tests (Bonferroni corrected) followed the two-way ANOVA. Assumptions of residual normality and homogeneity of variance were investigated using Shapiro-Wilk and Levene's tests, respectively. Normality and equal variance were confirmed. If data violated the sphericity assumption, Greenhouse-Geisser (*ε* < 0.75) or Huynh-Feldt (*ε* > 0.75) corrected values were reported.

A nonparametric Mann-Whitney *U* test for independent samples was used to compare Moebius and control group answers from the TEC-1. One Moebius subject did not complete the TEC-1 and was excluded from the analysis. Finally, we correlated the thermal values for each emotion condition with the TEC-1 scores. Data were analyzed by means of Statistica 8.0 (StatSoft, Tulsa, OK, USA).

## 3. Results

### 3.1. Thermal Data: Group Temperature Variations in relation to Conditions

A repeated measures ANOVA (3 × 2) was performed on the resampled variations of mean nasal tip temperatures. We did not find any differences between the two groups (*p* = 0.432). The results highlighted a significant effect of emotion condition (*F*_(1.53, 33.71)_ = 10.99; *p* ≤ 0.001^∗^; *η*^2^ = 0.325); post hoc tests showed that nasal tip temperature during the sadness condition significantly increased compared with that during the happiness (*p* = 0.013) and fear (*p* ≤ 0.001^∗^) conditions (for descriptive statistics, see Table 2). No significant difference was observed between the fear and happiness conditions (*p* = 0.133) (Figure 3(b)). The group × emotion condition interaction was not statistically significant (*p* = 0.447).

**Figure 4:**
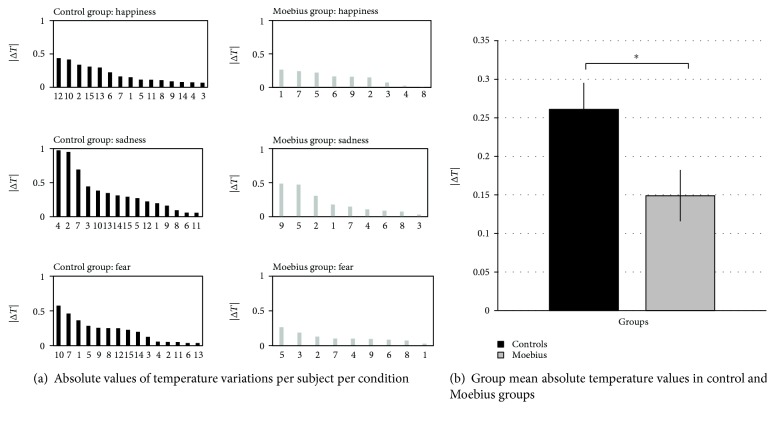
(a) Absolute value of the change in temperature from baseline per participant during each of the experimental conditions. Moebius participants exhibit a lower thermal modulation compared with control participants. (b) Group mean absolute temperature values in control and Moebius participants. Control participants have significantly more intense thermal modulation compared with Moebius participants. Means and standard errors (SE) are reported for both control and Moebius groups.

As shown in Figure 3(a), during all of the experimental conditions, Moebius participants exhibited a less appreciable thermal modulation compared to control participants while watching emotional stimuli. To measure any possible differences in the intensity of thermal modulation between the two groups, we considered the absolute value of change in temperature from baseline. As previously suggested, control participants exhibited a larger thermal response than Moebius participants during each experimental phase (Figure 4(a)).

A one-way ANOVA performed on the absolute value of the change in temperature from baseline revealed a significant effect of the group (*F*_(1, 22)_ = 4.732; *p* = 0.041; *η*^2^ = 0.177), with control participants having higher absolute changes in temperature (0.261 Δ*T*) than Moebius participants (0.149 Δ*T*) (Figure 4(b)).

### 3.2. Test of Emotion Comprehension (TEC-1)

**Figure 5:**
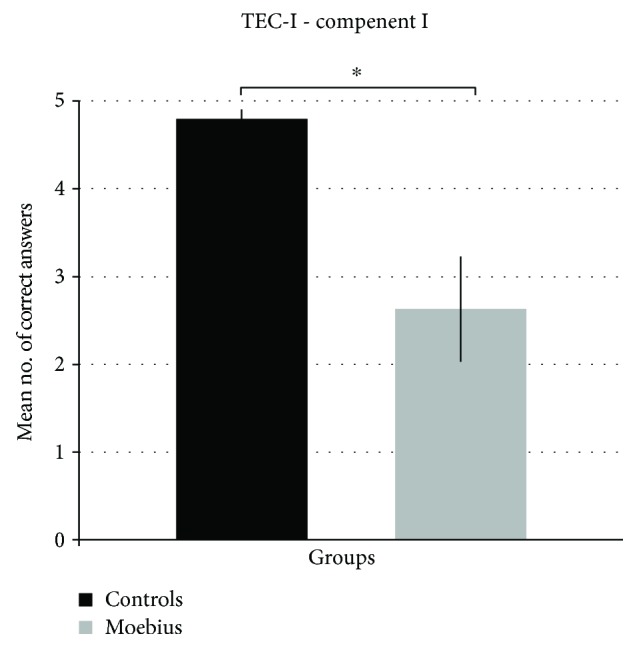
Mean number of correct answers from both control and Moebius participants. During the emotion recognition task (*TEC-1*), control participants performed better than Moebius participants. Means and standard errors (SE) are reported for both control and Moebius groups.

Mann-Whitney *U* tests were performed to assess if control and Moebius participants' scores significantly differed during the *Test of Emotion Comprehension* (TEC-1) administration. The results showed that the Moebius group had a lower level of facial emotion recognition (mean = 2.63; SD = 1.69) than the control group (mean = 4.80; SD = 0.41) (*p* = 0.002) (Figure 5). The autonomic responses and TEC-1 scores were not significantly correlated (*p* > 0.05).

## 4. Discussion

The purpose of our study was to detect psychophysiological responses in children affected by MBS by means of a fIRT camera. Moebius and control participants were asked to observe two sequences of emotional cartoon video stimuli representing three main emotions: happiness, sadness, and fear. Changes in nasal tip temperature were measured during the observation of the stimuli, and the results showed a significant difference between emotional conditions. Both MBS and control participants showed an increase in nasal tip temperature during the “sadness” condition [73], but Moebius participants were characterized by a less pronounced change in nasal tip temperature across all three of the experimental conditions. Recent studies investigating the ANS response specificity in emotion found a dual sympathetic-parasympathetic coactivation in response to “sadness” [73]. Several studies using video clip stimuli to induce feelings of sadness have found that crying is associated with sympathetic activation, while parasympathetic activation is typical of sadness without crying. Specifically, an activating sadness response (crying) appears to be typified by increased cardiovascular sympathetic response and changed respiratory activity, while a deactivating sadness response (noncrying) is distinguished by a decrease in sympathetic activation. Furthermore, noncrying sadness is characterized by decreased HR associated with decreased electrodermal activity [73]. These results are in line with our thermal findings. Although nasal tip temperature increased in both groups during the sadness condition, Moebius patients exhibited a generally weaker thermal response. The reason why it was not possible to highlight a significant differential response to emotional conditions between MBS and control participants is probably due to the interindividual variability of participants' thermal response to each emotion. For this reason, we considered the absolute values of participants' thermal responses (independently of the direction of thermal variation with respect to a neutral baseline) in order to identify differences between groups. Our data revealed a weaker, nonspecific thermal response of Moebius children while watching emotional stimuli, with respect to control participants.

The diminished temperature changes observed in Moebius patients could be ascribed to a minor modulation of the autonomic system in response to emotional stimuli. This differential intensity of thermal change could be interpreted in terms of the tight link between an action-perception mechanism, which contributes to sensorimotor simulation and to the process of recognition of others' emotions, and coordinated changes in the autonomic system which control visceral responses associated with emotions [23, 31, 87].

Neuroimaging studies have shown that the observation and production of emotional facial expressions activate similar networks of brain areas [26, 38]. More specifically, in addition to the temporo-parietal-frontal areas, which are the core of the action-observation network, other regions such as the amygdala, the ACC, and the anterior insula show an overlapping activation during both imitation and observation of emotional facial expressions [26]. These regions are involved not only in processing the emotional content of a stimulus but also in coordinating the physiological responses associated with the emotion [21, 22, 25, 38]. Electrical stimulation of the anterior insula in the monkey has revealed that this region is composed of several sectors which generate different autonomic responses and facial motor patterns when stimulated [21]. This strengthens the proposal of a strict link between the production of emotional facial expressions and the physiological modifications associated with experience of them. Our results suggest that the autonomic response related to the observation of emotional stimuli is reduced in children with congenital facial palsy. Previous brain imaging studies have provided support for the crucial role of corticolimbic circuits in the regulation of emotions [88]; however, so far, none has investigated the effects of the lack of peripheral feedback on autonomic responses to emotional stimuli.

Although this was not the main purpose of our study, we also wanted to assess children's explicit comprehension of the emotions expressed by video cartoons. The difficulty in acquiring these behavioral measures (e.g., participants' identification of the emotion depicted by the characters of the videos and participants' feelings during the presentation of the cartoons) led us to administer a less complex task in order to assess children's ability to explicitly recognize basic emotions. We therefore examined the emotion recognition ability in Moebius children by means of a standardized test, TEC-1. Compared to control participants, Moebius participants showed impairments on the emotion recognition task, with lower scores than healthy children of comparable age. These findings suggest that the impairment of facial muscles involved in the emotional display could affect not only the autonomic response but also facial expression recognition [47, 89, 90]. These results, though preliminary, are also compatible with the reverse simulation model, which proposes that the preservation of cortical control of the facial muscles is necessary to fully comprehend the emotional state of the other [35].

A few reports have tested the capacity of Moebius patients to recognize emotions, and the results are inconsistent [51, 53–55]. Most of these studies tested a small group of adult patients, with significant interindividual variability. One study utilized a considerable number of adult patients [54], and the authors did not find any evidence of facial emotion recognition deficits. However, it must be noted that this study suffers from some critical methodological limitations, such as the indirect assessment of participants' performance and of their neurological deficits.

Our study is the first to use a relatively large sample of very young patients to investigate the effects of facial muscle paralysis on both autonomic responses and emotion recognition. The investigation of these issues early in development is critical for the detection of emotional processing mechanisms at a stage where more complex cognitive strategies might not yet compensate for their deficits. In this regard, a large amount of literature has focused on how and when children's decoding of facial emotions develops [91, 92]. In the early stages of postnatal development, infants discriminate between different facial expressions and respond appropriately to different emotions displayed by their caregiver [93]. Furthermore, even if the debate revolving around the existence, prevalence, and meaning of neonatal imitation is still underway (see [94, 95], but also see a reexamination of this study [96] by Meltzoff and colleagues, which led to opposite results), much of the literature suggests that newborns are capable of mimicking certain facial expressions, such as smiles, indicating an early capacity to match own and others' facial expressions [96–99].

Considering that MBS facial paralysis is present since birth, we can hypothesize that MBS patients will exhibit mild deficits in the development of a fully functional MNS during the early stages of life. According to a theoretical developmental account [100, 101], after birth, facial expression synchronization with caregivers is critical to creating a link between the “self” and the “other” and to ensuring the shaping of the mirror mechanism supporting social communicative functions. Indeed, neonates are able to engage in reciprocal and emotional face-to-face interactions with their mothers. These exchanges, including facial and vocal expressions and gestures, are present immediately after birth and in the first month of life [102] and can be important for the development and function of the MNS [97, 103–105]. Recent studies have shown that based on such mother-infant face-to-face exchanges, the capacity of neonates to develop social expressiveness is related to their ability to produce appropriate emotional facial expressions and is correlated with the mother's skill in mirroring or marking such expressions [102, 105]. We do not know how this type of early experience could impact brain and emotional development, and this requires further investigations related to brain activity in cortical motor regions during mother-infant interactions in early development. However, children with MBS, due to their inability to express emotions through the face, might experience reduced quality of social interactions. It has been suggested that Moebius children might receive diminished facial responses from other individuals who, not perceiving a clear facial response during interactions, are less encouraged to socially engage and interact with them facially [106]. These hypothetical reduced inputs from both caregivers and other children, especially during early developmental periods, could have occurred from birth through childhood, resulting in an overall lower exposure to facial stimuli and consequent biased responses compared to healthy control participants.

Despite our findings that Moebius children have some deficits in recognizing emotions, they are still capable of understanding the emotional content of complex stimuli. The ANS response results, showing a similar, though less intense, thermal response in Moebius children compared with control participants, suggest that several cognitive processes may be used by Moebius subjects in order to understand the emotional content of complex stimuli. It is possible that although subtle aspects of emotion recognition are impaired as a consequence of altered facial mimicry, brain plasticity during development and the exploitation of other cognitive strategies could be employed by Moebius patients to compensate for the early deficits.

At this point of the discussion, it should be mentioned that the role of the MNS in action understanding has been debated and discussions are still ongoing (see [107, 108], but also see [32, 109]). According to Hickok [110], action understanding and motor system function could be dissociated. In contrast to this view, a meta-analysis found impairments in recognizing actions associated with lesions in MNS regions [111]. These results are further supported by a study by Michael and colleagues [112] where participants received theta-burst stimulation to temporarily create a lesion on the premotor cortex, causing clear impairments in understanding actions performed by others. Our study does not allow us to support the hypothesis that facial mimicry is the only process involved in emotion understanding; in fact, other mechanisms could be exploited when automatic peripheral facial feedback is absent. However, a diminished autonomic response in Moebius patients makes us propose that, in line with embodied theories [48], facial mimicry could represent a key mechanism for emotional processing.

A few methodological limitations of our study should be mentioned. Cartoon stimuli differed in length because of our specific aim to present participants with an authentic content able to induce a particular emotion. Since emotional content is the actual variable expected to influence thermal values, the differential duration of the stimuli alone would not have affected the thermal results, given the slow dynamic of thermal response. This is further confirmed by our main result showing a difference between the experimental and control groups that was independent of stimuli duration.

Additionally, Moebius patients' impaired ocular abduction could be considered one limit of the current study; however, as discussed by Carta and colleagues [113], these patients compensate for their lack of lateral version with large movements of the head.

It has also to be pointed out that the extreme rarity of the syndrome, the limited age range taken into account, and the exclusion of patients with autism or mental retardation let us to include only a limited number of participants (9 participants), which did not permit further analysis. Despite the challenges involved in acquiring a sample large enough to study this syndrome, it would be worthwhile for future studies to explore the emotion recognition ability at different ages and/or gender differences.

Lastly, although fIRT is at the forefront of the techniques allowing ANS recording in a naturalistic setting, the thermal signal as a result of perspiration and muscle activity and the time course of metabolic responses are rather sluggish. Nevertheless, the reliability and feasibility of fIRT have been confirmed by several comparisons with other standard methods of ANS measurement such as electrocardiography (ECG) and skin conductance or galvanic skin response (GSR) [70]. As this technology is still in development, there is a need to determine if heat patterns indicate discrete emotions [114] or dimensional responses [115]. It would therefore be useful to integrate this method with other techniques to compare ANS measurements within the same experimental paradigm.

## 5. Conclusions

MBS patients' decreased capacity to activate a motor simulation process during the decoding of emotions could have led to a diminished thermal variation and ANS response during the observation of complex emotional stimuli. It is possible that patients' impairments in mimicking could have affected not only their cognitive emotion recognition processes but also the way in which they are related to ANS changes associated with emotions. If the absence or reduction of motor representations resulted in deficiencies in their early facial expression recognition mechanism (as a consequence of having limited control of facial muscles), MBS individuals might learn during development to cognitively deduce the emotional states of others by using a number of visual cues related to the face and the environmental context [116, 117]. By exploiting such cues, MBS patients can extract regularities and develop conceptual knowledge of an emotion [89]. Further studies are crucial in order to address the relationship between the level of emotion recognition deficits and the magnitude of the autonomic response, in order to better understand their possible causal relationship.

## Figures and Tables

**Table 1:** Moebius subjects' medical cases. The term “laterality” refers to the kind of facial paralysis that can be unilateral or bilateral; the sixth and seventh cranial nerves are usually involved, but other nerves may also be affected. “Associated pathologies” linked to Moebius syndrome can involve possible hand and foot anomalies, muscle hypotonia, hypoacusis, swallowing and speech problems, and Poland syndrome.

ID no.	Sex	Laterality	Cranial nerves involved	Additional functional deficits and associated pathologies
1	M	Unilateral left	VI, VII	—
2	M	Bilateral	VI, VII, III, IV	Strabismus, hypotonia, hypoacusia of the right ear, speech deficit (articulation-phonetic disorders), right plagiocephaly, psychomotor delay, epileptic seizures, cardiac crisis
3	F	Bilateral	VI, VII, XII	Foot malformations
4	F	Unilateral left	VI, VII, XII	Speech deficit, club feet
5	M	Bilateral	VI, VII, XII	Club foot, brain stem atrophy with enlargement of the fourth ventricle, hand deformities
6	F	Unilateral right	VI, VII, XII right	Micrognathia, tongue hypoplasia
7	F	Bilateral	VI, VII	Bilateral mixed hypoacusia, hypotonia, delayed growth, laryngomalacia, palatal schisis, coloboma of the right optic nerve
8	M	Bilateral	VI, VII, XII left	Respiratory difficulties, micrognathia, hypotonia, psychomotor delay, club foot
9	M	Bilateral	VII	No ocular deficits, speech delay

**Table 2:** Descriptive statistics for each group and condition.

	Group	Happiness	Sadness	Fear
Mean	Control	0.199	0.364	0.216
	Moebius	0.144	0.212	0.118
Std. error mean	Control	0.033	0.075	0.042
	Moebius	0.030	0.057	0.023
Standard deviation	Control	0.128	0.290	0.163
	Moebius	0.090	0.171	0.069
Variance	Control	0.016	0.084	0.026
	Moebius	0.008	0.029	0.005

## Data Availability

The data used to support the findings of this study are available from the corresponding author upon request.
